# Domestic Summary

**Published:** 1839-10-05

**Authors:** 


### DOMESTIC SUMMARY.

*Institution of the Pathological Society of Philadelphia.*—A society has recently been organized in Philadelphia under the above title. Its objects are, first, the exhibition of specimens of morbid anatomy, which are met with in hospital and private practice; and, secondly, the collection and preservation of these specimens in a museum of pathological anatomy. The Society has already gone into operation, and holds weekly meetings. We shall, from time to time, offer reports of the more interesting proceedings of this very useful society, and, in the mean time, we give below a list of the officers.

*President*—W. W. Gerhard, M. D.

*Vice Presidents*—C. W. Pennock, M. D., T. Stewardson, Jr., M. D.

*Secretary*—George W. Norris, M. D.

*Treasurer*—Edw. Peace, M. D.

*Curators*—Paul B. Goddard, M. D.; W. Pepper, M. D.; B. F. Hardy, M. D.

---

*Jefferson Medical College.*—Professor Samuel McClellan having retired from the faculty of this institution, Professor R. M. Huston has been transferred from the chair of Materia Medica to the vacant chair of Midwifery, and the former chair has been merged in that of Professor Dunglison.

---

*Western Journal of Medicine and Surgery.*—The Western Journal of the Medical and Physical Sciences, published at Cincinnati, under the principal editorship of Dr. Drake, has been suspended. Dr. Drake has transferred the subscription list to a new journal, proposed to be established at Louisville, Kentucky, under the title of the Western Journal of Medicine and Surgery, which takes the place of the Louisville Journal of Medicine and Surgery, suspended in the beginning of the current year. The *Western Journal* will be published monthly, the first number to be issued January, 1840.

